# Hospital Staff Perspectives on the Drivers and Challenges in Implementing a Virtual Rehabilitation Ward: Qualitative Study

**DOI:** 10.2196/54774

**Published:** 2024-06-27

**Authors:** Leanne Greene, Miia Rahja, Kate Laver, Vun Vun Wong, Chris Leung, Maria Crotty

**Affiliations:** 1 Rehabilitation, Aged and Palliative Care, Flinders University, Adelaide, Australia; 2 Division of Rehabilitation, Aged and Palliative Care, Southern Adelaide Local Health Network, Adelaide, Australia

**Keywords:** gerontology, geriatric, geriatrics, older adult, older adults, elder, elderly, older person, older people, ageing, aging, aged, telerehabilitation, rehabilitation, rehab, workflow, hospitalization, health services accessibility, accessibility, clinical decision-making, equipment failure, telemedicine, telehealth, tele-medicine, tele-health, virtual care, virtual health, virtual medicine, remote consultation, telephone consultation, video consultation, remote consultations, telephone consultations, video consultations, personnel, hospital

## Abstract

**Background:**

Over the past decade, the adoption of virtual wards has surged. Virtual wards aim to prevent unnecessary hospital admissions, expedite home discharge, and enhance patient satisfaction, which are particularly beneficial for the older adult population who faces risks associated with hospitalization. Consequently, substantial investments are being made in virtual rehabilitation wards (VRWs), despite evidence of varying levels of success in their implementation. However, the facilitators and barriers experienced by virtual ward staff for the rapid implementation of these innovative care models remain poorly understood.

**Objective:**

This paper presents insights from hospital staff working on an Australian VRW in response to the growing demand for programs aimed at preventing hospital admissions. We explore staff’s perspectives on the facilitators and barriers of the VRW, shedding light on service setup and delivery.

**Methods:**

Qualitative interviews were conducted with 21 VRW staff using the Nonadoption, Abandonment, Scale-up, Spread, and Sustainability (NASSS) framework. The analysis of data was performed using framework analysis and the 7 domains of the NASSS framework.

**Results:**

The results were mapped onto the 7 domains of the NASSS framework. (1) Condition: Managing certain conditions, especially those involving comorbidities and sociocultural factors, can be challenging. (2) Technology: The VRW demonstrated suitability for technologically engaged patients without cognitive impairment, offering advantages in clinical decision-making through remote monitoring and video calls. However, interoperability issues and equipment malfunctions caused staff frustration, highlighting the importance of promptly addressing technical challenges. (3) Value proposition: The VRW empowered patients to choose their care location, extending access to care for rural communities and enabling home-based treatment for older adults. (4) Adopters and (5) organizations: Despite these benefits, the cultural shift from in-person to remote treatment introduced uncertainties in workflows, professional responsibilities, resource allocation, and intake processes. (6) Wider system and (7) embedding: As the service continues to develop to address gaps in hospital capacity, it is imperative to prioritize ongoing adaptation. This includes refining the process of smoothly transferring patients back to the hospital, addressing technical aspects, ensuring seamless continuity of care, and thoughtfully considering how the burden of care may shift to patients and their families.

**Conclusions:**

In this qualitative study exploring health care staff’s experience of an innovative VRW, we identified several drivers and challenges to implementation and acceptability. The findings have implications for future services considering implementing VRWs for older adults in terms of service setup and delivery. Future work will focus on assessing patient and carer experiences of the VRW.

## Introduction

Implementation rates of virtual wards have increased over the last decade, mainly driven by technological advancements and the COVID-19 pandemic [1-5]. The UK National Health Service (NHS) report that virtual wards, including hospital-at-home services, are a safe and efficient substitute for inpatient care that is facilitated by technology [2,6,7]. Virtual wards offer at-home acute care in the form of monitoring and treatment to individuals who would otherwise require a hospital bed, using a flexible combination of remote and in-person services [2,6]. As virtual models of care are relatively novel, there can be ambiguity around terminology [2,8]. In the NHS model, virtual wards for older individuals function akin to hospital-at-home services, primarily delivering care through face-to-face interactions [8].

Virtual wards aim to prevent avoidable hospital admissions, facilitate early discharge home, and increase patient satisfaction [3,6,9]. For the older adult population, hospital admissions carry potential risks, including deconditioning, delirium, and hospital-acquired infections [10], so opting for home-based treatments may be beneficial [11]. Consequently, significant investments are being directed toward the expansion of virtual care models in health care systems, such as the UK NHS, encompassing patients with frailty [12,13]. However, economic assessments of virtual models often fail to meet quality criteria, leading to varying estimated cost savings [2].

The implementation of virtual care models has been hindered by issues such as nonadoption, abandonment, and difficulties with scaling up, particularly if the model requires significant changes to the broader care system [14-16]. There is a paucity of research investigating the sustainability of virtual models [15], particularly virtual wards [2]. Despite substantial policy-level discussions and modest proof-of-concept studies, virtual health care models are seldom mainstreamed [17-19]. The success or failure of implementing innovative virtual health care models is often attributed to a complex combination of facilitators and barriers, rather than individual factors alone, such as time pressures, infrastructure, unreliable equipment, and staff and service user preferences [20]. Understanding these issues is important as virtual care marks a monumental change in the delivery of health care for older individuals [2].

In practice, virtual wards are often added to existing hospital services as a solution to a bed capacity problem rather than being designed from the ground up as new freestanding services [21-23]. When hospital-based staff are asked to establish a virtual ward and commence providing services through videoconferencing and monitoring, significant shifts in practice are required. The facilitators and barriers experienced by hospital staff for the rapid implementation of these novel care models are not well understood, as evidenced by the paucity of literature in the area. There is also a lack of guidance for the provision of virtual wards, with calls for information on how these new models of care are being implemented [8]. This paper addresses a gap in the existing literature by offering insights into the experiences, perceptions, and attitudes of hospital staff working within a newly established Australian virtual rehabilitation ward (VRW). To our knowledge, no previous studies have delved into this specific research area. The implementation of the VRW was undertaken by the Flinders Medical Centre, part of the Southern Adelaide Local Health Network (SALHN) in South Australia, in response to the increasing demand for programs aimed at preventing hospital admissions. We explored the facilitators and barriers of the VRW from the view of staffs and present reflections for service setup and delivery.

## Methods

### Design

**Figure 1.**
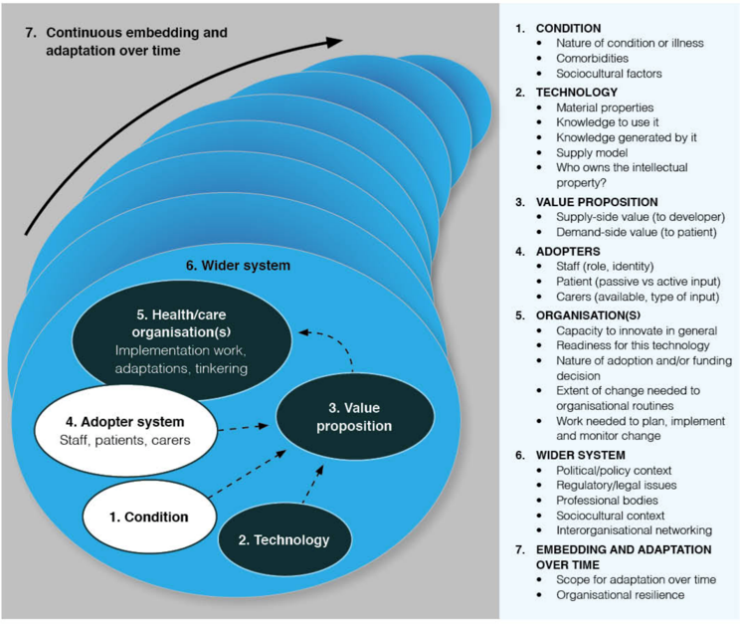
The Nonadoption, Abandonment, Scale-up, Spread, and Sustainability (NASSS) framework, first published in 2017, provides a structured approach to examine the factors that impact the adoption, nonadoption, abandonment, spread, scale-up, and sustainability of health care technology (reproduced from Abimbola et al [27], which is published under Creative Commons Attribution 4.0 International License [31]).

A multidisciplinary research team (clinicians and academic researchers) conducted this study under a constructivist paradigm [24]. Data were integrated and analyzed using the Nonadoption, Abandonment, Scale-up, Spread, and Sustainability (NASSS) framework [15] to understand staff experiences (Figure 1). Interview questions focused on the 7 domains of the NASSS framework including condition, technology, value proposition, adopters, organizations, wider system, and embedding and adaptation over time. These domains provided an analytical framework for organizing, classifying, and contrasting staff experiences into a rich narrative. The NASSS framework was chosen as it was designed to evaluate technology-supported change projects in health or social care [15] and, therefore, fitted with the aim of our research. Moreover, it has been previously used to evaluate technology-supported health care programs [25-27]. Other frameworks such as the Reach, Effectiveness, Adoption, Implementation, and Maintenance (RE-AIM) framework [28]; Precede-Proceed model; Dynamic Sustainability framework; or the Practical, Robust Implementation and Sustainability Model (PRISM) [29] were not chosen as they lacked the technology focus of the NASSS framework. We also report our study according to the COREQ (Consolidated Criteria for Reporting Qualitative Research) guidelines [30] to improve the quality and transparency of our work.

### Ethical Considerations

Ethics approval was received from the SALHN (Southern Adelaide Clinical Human Research Ethics Committee; 2022/HRE00107). Written informed consent was obtained from all participants, who were informed of their right to withdraw from the study at any time. The data presented in this article have been deidentified. Participants did not receive any compensation.

### Setting

South Australia spans a geographical area of 983,482 km^2^ with a population of 1.8 million [32]. SALHN services a community of approximately 400,000 people in the southern metropolitan area of Adelaide. The population in Southern Adelaide skews toward older age groups compared to other areas of Adelaide and the broader Australian population, with a projected accelerated aging rate [33]. The prevalence of lone-person households, concentrated among older age groups, is rising, and these demographic shifts will likely amplify the demand for health care services [33]. The Flinders Medical Centre is the second-largest tertiary hospital in Adelaide with nearly 600 beds, offering a wide range of medical, surgical, obstetric, and pediatric services.

### Virtual Rehabilitation Service

The virtual rehabilitation ward (VRW) provides acute clinical care through rapid assessment and rehabilitation to patients with a range of diagnoses in their own homes. The service provides an alternative to hospital-based rehabilitation and allows patients to be discharged earlier from inpatient wards. A team of multidisciplinary staff (see Table 1), including doctors, nurses, and allied health professionals, work with the individual to achieve rehabilitation goals via a mix of tailored video calls and home visits over a 2-week period with daily clinical reviews. The patient’s medical status is also monitored remotely, for instance, blood pressure, oxygen saturation, temperature, and weight. All equipment is loaned to the patient (eg, iPad with cellular connection via SIM card and monitoring equipment), and training on how to use the equipment is provided by care staff. Home visits can occur as required, and an initial visit is made to set up equipment and provide training.

**Table 1.** Virtual rehabilitation ward workforce structure.

Profession	Full-time equivalent (hours)
Medical consultant, registrar, and resident medical officer	2.8
Nursing (clinical and enrolled)	10.5
Allied health professionals (physiotherapist, occupational therapist, pharmacy, psychology, exercise physiologist, social worker, speech pathologist, dietetics, and allied health assistants)	16

The service is available to individuals over the age of 18 years, but most patients are older adults. Between the commencement of the service in January 2022 and August 2022, a total of 181 (79%) out of 229 patients were aged 65 years or older, with an average age of 73.9 years and a median age of 75.0 years. Local hospital ward staff refer patients to the VRW, and a VRW coordinator assesses the individual’s suitability for the program, usually in person while they are still an inpatient. The VRW accepts a wide range of patients with complex care needs, including individuals recovering from trauma, patients undergoing cancer rehabilitation, or those who require postsurgery care. The service delivers time-limited interventions and monitoring (usually 2 weeks) based on clinical needs. The service runs 7 days a week 24 hours a day, with full staffing between 8 AM and 8 PM and access to an on-call doctor available outside these hours. Patients are provided with a telephone number to contact if their symptoms worsen. The VRW is supported by a contracted external telecommunication provider, which supplements internal SALHN digital health support.

### Participant Recruitment

Between July and September 2023, staff previously (ie, rotational junior doctors) or presently employed by the VRW were invited to participate in a semistructured interview exploring the implementation of the VRW. In phenomenological studies, a purposive sampling strategy—in this research, maximum variation sampling—is supported to recruit participants who have experienced the phenomenon under study. We aimed to recruit a diverse range of health care staff (ie, clinical, administration, and information technology) to capture various perspectives on the topic of interest, thereby illuminating diversity and revealing patterns or commonalities in traits across the spectrum [34-38]. Recruitment occurred through the ward managers circulating research information via email and word of mouth. Overall, 21 interviews were conducted, and participant characteristics are presented in Table 2. Data collection was ceased when all the staff who wanted to take part in the study had been interviewed. The number of VRW staff was smaller compared to conventional inpatient rehabilitation wards (Table 1). A total of 9 staff members declined because they were not interested in taking part in research or because they did not have the time to take part in an interview. No participants withdrew from the research after consenting. Each participant was interviewed only once, and no relationship was established prior to study commencement.

**Table 2.** Participant demographics (N=21).

Demographics		Value
**Occupation, n (%)**		
	Doctor	6 (29)
	Nurse	3 (14)
	Physiotherapist	3 (14)
	Exercise physiologist	1 (5)
	Occupational therapist	1 (5)
	Information technology officer	2 (10)
	Administrative assistant	1 (5)
	Manager	2 (10)
	Social worker	1 (5)
	Pharmacist	1 (5)
Age (years), mean (range)		39.1 (25-62)
**Sex, n (%)**		
	Female	15 (71)
	Male	6 (29)

### Qualitative Data Collection and Analysis

We used phenomenology to understand the meaning of the perspectives of staff who worked on the VRW [37]. A researcher independent of the health service conducted the semistructured, audio-recorded interviews (LG). Field notes were made during the interviews to aid reflexivity [39]. A total of 20 interviews were in person and 1 was conducted virtually via Microsoft Teams. To ensure convenience for the staff, in-person interviews were held in a meeting room on the same floor of the hospital as the VRW offices. For the virtual interview, both the researcher and the participant were in their respective homes. Interviews lasted between 45‐60 minutes and no one else was present besides the participants and researcher. Interview questions and descriptions of the 7 NASSS domains are provided in Multimedia Appendix 1. Questions were tailored for each staff discipline, for example, doctors were asked about remote prescribing while allied health professionals were asked about remote therapy. We did not conduct a pilot test for this piece of research.

Deidentified, audio-recorded interviews were transcribed verbatim by a professional transcription service. One participant requested for their transcript to be returned to them for comment, but no amendments were made. The data were analyzed by 2 coders (LG and MR) using framework analysis [40] to identify the key themes and meanings that emerged from the participants’ descriptions. This method was chosen as it provides a rigorous and transparent approach for researchers to analyze multidisciplinary health research [41]. Moreover, as framework analysis is not aligned with a specific epistemological, philosophical, or theoretical perspective, it adapted well to the use of a preexisting theoretical framework (deductive analysis) while allowing room for revisions with inductive aspects of analysis [40]. In brief, the process of framework analysis involves organizing significant themes and issues into 5 distinct stages: becoming familiar with the data; identifying a thematic framework; indexing; charting; and finally, mapping and interpretation. Multimedia Appendix 2 [15,41-46] provides a detailed description of the methodology. NVivo 12 (QSR International) was used for coding and indexing the data into the 7 NASSS domains. The information was subsequently condensed and organized into a matrix. Multimedia Appendix 3 provides a description of our coding tree.

## Results

### Overview

Due to the small size of the VRW staff, where sometimes only 1 member represented each discipline, to ensure anonymity within quoted content, we have grouped them. Physiotherapists, exercise physiologists, occupational therapists, and social workers are grouped under the collective name “Allied Health Professional” and managers and administrative staff are grouped under “Leadership/Admin Team.” Two participants provided feedback on the findings via email and during an informal face-to-face meeting.

In summary, the VRW sought to serve patients with varied health conditions. Challenges arose in managing complex cases such as heart failure and cognitive impairments, impacting staff confidence in virtual care delivery. Despite technological benefits such as remote monitoring, interoperability issues persisted, hindering adoption. The service’s value lay in offering choice and access to care, particularly benefiting rural communities. However, risks included communication challenges with community care teams and less intensive therapy compared to inpatient settings. The transition to virtual care posed workflow and responsibility challenges, highlighting the need for staff training and support. Ambiguity surrounding the service’s identity and referral processes impacted resource allocation and patient expectations. Challenges in patient transfer and continuity of care were observed, along with resistance to hospital readmission and overcrowded emergency departments. Staff recognized the service’s potential but emphasized the need for specialized planning and ongoing adaptation. Adaptive actions included refining technology and identifying areas for improvement in patient care and service delivery.

### Domain 1: The Condition

The VRW services patients with several health conditions, including ones that require high-level care (Multimedia Appendix 4). Participants commented on how certain conditions, such as heart failure, fluid retention, and complex wounds, were more challenging to manage using a virtual ward approach in comparison to a traditional inpatient setting.

We’d have fluid overloaded patients, and you just don’t know how much they’re drinking, or you can’t do the same monitoring as you can in hospital.

[Doctor]

Comorbidities (including cognitive impairments, polypharmacy, frailty, disability [eg, limb impairments and tremors], and sensory impairments) were also discussed by all participants as factors impacting how confident the staff felt in engaging patients in the virtual ward service. There was a sense that the service might be better suited for patients with minimal cognitive impairment who are willing to engage with technology.

In the right population, yes. I think again, if they’re cognitively not good, or they’re really not wanting to engage through technology, then it’s very difficult. But yeah, I think if they’re willing to engage in that, I think it’s no different to being in the room with them.

[Allied Health Professional]

The service’s suitability was also influenced by sociocultural factors such as living arrangements. For example, the staff reported relying on carers to assist with virtual sessions or remote monitoring. Therefore, living alone or residing in a care home could potentially pose challenges.

I think it depends on what support the person has…So if they’ve got someone else there with them who can use the technology that works really well, and there’s definitely no issues there.

[Allied Health Professional]

### Domain 2: The Technology

A lack of interoperability between hardware and software systems was discussed by all 21 participants. There was agreement that the integration between different health care systems, service providers, technology, and security was poor and impeded adoption (Multimedia Appendix 4). This complexity appeared to make it difficult to pinpoint the exact sources of problems when difficulties occurred. Additional challenges came from unforeseen software updates, with the equipment dispersed across patients’ homes (Multimedia Appendix 4).

It’s like, “Don’t really care whose fault it is, can someone just fix it?”

[Allied Health Professional]

The remote monitoring equipment and the capability to make video calls was perceived as advantageous, since it has assisted staff in monitoring patient conditions and facilitating the escalation of care or transferring to another health care service when necessary. This was particularly commented on by doctors, although other staff also made reference to the equipment’s benefits in escalation. Despite staff’s efforts to streamline the process for simplicity, there were reliability and usability concerns, occasionally affecting rapport (Multimedia Appendix 4).

...it’s not 100% reliable for us, and for us to escalate care of patients based on an unreliable system is difficult to do…We need to have technology that we can rely on, that is safe.

[Leadership/Admin Team]

There was, however, recognition that some of these technology failures could stem from a lack of knowledge and education among both staff and patients (Multimedia Appendix 4).

...maybe we don’t facilitate the education as good as we should. We are out there for what, maybe half an hour. And then, “Here’s your kit, off you go.”

[Leadership/Admin Team]

Medtasker, a mobile communication and task management platform, was widely used and commended for its ability to minimize work duplication and reduce the need for excessive phone calls and emails.

Because the virtual team is sort of here mornings, evenings, there might be people that might have seen the patient a few days and then they’re not the next and so forth. We’re trying to work out the best ways of managing communication. And I think a program like Medtasker helps.

[Doctor]

### Domain 3: Value Proposition

The value of the service was the choice it provided patients on where they received their care (Multimedia Appendix 4). The staff felt that home-based treatment, especially for older individuals, would be a preferred and more comfortable option than staying in hospital. The service was considered empowering and enabled access to care for rural communities who might not be able to access rehabilitation services otherwise (Multimedia Appendix 4).

I think the gaps that it bridges is amazing and huge, because our country patients, who aren’t able to travel…I see cancer patients who are palliative who really benefit from that ability to be able to connect via video link.

[Doctor]

The value of the VRW for patients was also discussed by identifying risks. The opinions about this varied, but in general, there was a sense that the VRW did not pose more risks, just different risks. One concern, particularly with doctors, was that patients often resumed contact with their community care teams (general practitioners [GPs] and community-based medical specialists).

…managing patients who are still attending their GPs, their specialists in other hospitals in the background, and if you do not have an understanding of what there is happening there and you are involved, I think that is somewhere where some confusion can happen.

[Doctor]

Additionally, there were fewer opportunities for physical evaluation, monitoring, and therapy, leading to concerns that clinicians might miss early signs of deterioration. This concern was elaborated upon by 4 of the interviewed doctors, while nurses and allied health professionals also indicated unease about not being able to examine severely ill patients as thoroughly as they typically would on a ward. Allied health professionals discussed patients having less-intense therapy compared to inpatient wards (Multimedia Appendix 4).

...a lot of clinical signs we aren’t able to pick up by a video link, so we’ll assess them a particular way, but then when the registrar’s gone and done a home visit just because we were worried for whatever reason, we’ve actually found other signs which have triggered escalation of care later, which we weren't able to identify through video links.

[Doctor]

### Domain 4: The Adopters

The commencement of the VRW has imposed many changes to staff practices. The interviews delved into the need for a cultural shift and how some participants (and their colleagues) were resistant to adopting the virtual approach. Face-to-face patient interactions were preferred by many, either because they were accustomed to it or because they perceived clinical advantages in such treatments. There was a tendency for conducting initial meetings in person, followed by remote reviews, whenever feasible.

That’s just the way that I've nursed for 30 years…I’m not used to trying to do that over a screen.

[Nurse]

There appeared to be some uncertainty surrounding workflows and professional responsibilities, leading to concerns that clinical staff might be required to assume technology or administrative duties (Multimedia Appendix 4).

We’re clinical, we’re not IT.

[Leadership/Admin Team]

As the service developed, the participants described how they have acquired valuable knowledge about the essential support, training, and resources needed. For instance, the team has recently gained a pharmacist, and this addition has helped alleviate previous prescription medication challenges. Furthermore, due to the unanticipated complex care needs of the referred patients, it has been crucial to have experienced staff to handle the workload effectively. As such, the clinical team has made staffing adjustments to accommodate for the large number of junior staff working within the service (Multimedia Appendix 4). It was highlighted that for a virtual ward service to succeed, the staff should possess strong clinical skills to ensure adaptability and flexibility in care delivery (Multimedia Appendix 4).

…just the more experience and the more training and the more feeding off each other and learning of each other, the more we’re adapting the telehealth. There is a huge education component that’s needed for junior staff and a lot of support for people that aren’t experienced.

[Allied Health Professional]

A shift in the burden of care to the patient and their family was also discussed. Staff particularly recognized the essential role that carers play in the patients’ journey during virtual care.

...we heavily use carers and family in this model because not only can they help a lot of the time with setting up the technology and working it out and things like that, they’re quite often present during the reviews and we quite often use them to just be our hands.

[Allied Health Professional]

### Domain 5: The Organization

There was a sense of ambiguity surrounding the identity of the service both for the staff and patients. The service was set up to provide rehabilitation with a primary focus on functional recovery, but hospital pressures resulted in large numbers of referrals from acute wards, leading to more medically complex referrals than the team had anticipated. This presented a particular challenge for allied health professionals who felt that their roles became almost redundant for certain patients, such as those from oncology wards with newly diagnosed conditions that they were struggling to come to terms with.

We have a lot of pal[liative] care patients coming through lately. And just the questions that they ask, and the difficult conversations, and where to go with rehab. Because it’s not really restorative, it’s more maintenance and optimizing.

[Allied Health Professional]

Participants expressed frustration with the intake process, as they believed that referrals should involve patients who are willing and able to use technology as part of their rehabilitation journey.

There needs to be more done in the triage of people’s capacity to use technology…we’ve been getting people who’ve just said to us, “I’m not using the iPad.”

[Allied Health Professional]

There was a perception that patients were not accurately informed about the service or that some patients did not fully understand the implications of the term “Virtual Rehabilitation Ward.” Patients often had unrealistic expectations, assuming that rehabilitation at home would be less intensive than in a traditional ward setting (Multimedia Appendix 4).

...they would think that it’s a ward, but then the virtual part of it, sometimes, well some people don’t know what that means. Some people think it’s purely virtual so that there’s no home visiting service.

[Leadership/Admin Team]

The importance of resource allocation and the environmental setup was also discussed. Given the rise in the complex patient population requiring more in-person care, vehicle access was often an issue, particularly for nursing and allied health care staff (Multimedia Appendix 4). Open-plan offices and a lack of private spaces for confidential calls were among the challenges discussed (Multimedia Appendix 4). Staff emphasized that the organization and setup of the service were crucial factors in its successful adoption, stressing the need for a balanced approach that offers a supportive office environment and has enough room and resources to deliver care flexibly.

Because you want to have the team together and interacting like they would on ward in a way because they have that, there’s then that team camaraderie but also that informal sharing of knowledge and teaching each other…but you also need space to be able to operate and talk to patients and hear them.

[Doctor]

### Domain 6: The Wider Context

Staff felt that the new service aimed to fill gaps in the existing health care system, but it lacked a clear pathway structure and well-defined boundaries.

...a lot of people are starting to get frustrated because there’s just no clear pathway structure, anything of what we are and what we’re meant to be doing and where we can set those boundaries of that’s not our role. It just seems to be like take everything, deal with it when it’s there.

[Allied Health Professional]

All staff members were mindful that patients are eager to leave the hospital and might be willing to accept any form of home service. They were also aware of the hospital’s pressure to discharge patients promptly due to the scarcity of available beds.

I think so many of them are just so desperate to get out of hospital they’ll agree to anything. And I think that the hospital is so desperate to get them out of hospital as well that they will sign them up to whatever program will take them.

[Leadership/Admin Team]

The wider context, especially in terms of the continuity of care, was discussed from service and interorganizational perspectives. Staff emphasized the importance of connecting with GPs before a patient’s discharge to ensure the continuity of care; however, this was rarely done in practice.

Especially with our frailty pathway, we’re meant to call the GP and talk about our frailty management plan once they're discharged from our service, but that’s just been really difficult. Haven’t been able to get through to the GP.

[Allied Health Professional]

Support and knowing how to connect patients with services following discharge were also discussed as a matter of importance.

…discharging patients from inpatient rehab, there’s only a couple of go-to places, but then it’s very different if patients are already out in the community. Because there’s a lot of options for further support that you can link them with.

[Doctor]

Transferring patients back to the hospital when their health deteriorated presented challenges. Staff encountered resistance from patients who were reluctant to return to the hospital setting. Moreover, escalation pathways were impeded by overcrowded emergency departments and a shortage of available beds in the hospital. These issues were encountered by all clinical staff.

The difficulty has been once we identify they need to come in, what pathway they go towards. ED’S ramping, there’s no beds in [local hospital], and then you’ve got to take those measured risks.

[Doctor]

### Domain 7: Embedding and Adaptation Over Time

In general, all of the staff recognized the advantages that the service could provide to clinicians, patients, and the broader health care system (Multimedia Appendix 4). Nevertheless, they emphasized the need for planning that considered the distinctive and specialized requirements of virtual wards.

We try and introduce new models like technology and you’re trying to just do it on the edge of everything else that is happening but really it needs its own setup…that’s one of the challenges really, is trying to introduce a new model of care into an old system.

[Leadership/Admin Team]

Staff were engaged with adapting and embedding the technology within the service in response to their patient’s needs. There was recognition that the service is in its early stages of development (Multimedia Appendix 4), and as a result, certain aspects are not yet finely tuned. While some staff regarded glitches as failures, many understood that creating a new service is a gradual process that demands refinement over time.

It’s changing that mindset, not seeing it as a failure. It’s just seeing it as a sidestep again for now…They see that as a failure, which it isn’t. Sometimes we’ve just got to fine tune things a little bit to be able to safely manage you at home.

[Nurse]

Sensemaking and collective reflection were evident throughout the interviews, with strengths of the service being the implementation of Medtasker as a platform for team communication and accountability, and remote health monitoring to help clinicians escalate care. Adaptive action is ongoing to ensure that the technology is more dependable and the service is reaching its target patient cohort. The team is continually learning and identifying areas for adaption: for example, having a stock of common medications to trial patients on them, and having more technology applicable to an older cohort with frailty such as fall devices, echocardiograms, and telemetry.

## Discussion

This paper addresses the knowledge gap related to the facilitators and barriers experienced by hospital staff during the implementation of a new VRW [2]. The service aimed to provide early discharge with a functional and medical care program for adults who are able to rehabilitate at home. The perspectives of staff are pivotal as previous research has suggested that clinician endorsement can account for a significant portion of the variability in the adoption, growth, and continuity of telehealth services [47-49].

The VRW team successfully managed complex patients in the community but highlighted how some conditions were more challenging than others, such as individuals with fluid retention or severe wounds. This was particularly pertinent for older individuals, especially those with comorbidities and those who reside alone or in care homes, where they might lack the capacity to manage monitoring and communication equipment. Older people who were not proficient with digital technology were not disadvantaged in terms of quality of care or through digital exclusion [2], as the team was flexible in providing tailored care and would provide in-person services. Yet, it appears that for a virtual ward to achieve success, the service must possess a clear identity that is supported by its triage and intake process. A mismatch was described between staff expectations of medically stable patients focused on rehabilitation and the actual referrals of frail patients of varied case mix who were discharged early in response to hospital pressures. These findings underscore the importance of the consideration of patient characteristics prior to enrollment and the necessity of tailored care.

Regarding technology, interoperability was poor, a common issue in virtual care [50,51], and there were delays in pinpointing problems that impeded adoption. Nevertheless, some of these challenges appeared to be rooted in a lack of staff and patient training on correct technology use, an issue that appears common in new technology-supported health care models [15,16]. The brevity of the VRW program (typically 2 weeks) posed challenges, as there is limited time available for staff to adequately train patients, particularly older individuals who may need more support [52]. The substantial effort required may also seem disproportionate to the program’s short duration. The attitudes around technology were multifaceted. While there was a consensus that the technology was useful, particularly in the escalation of care, promotion of self-management, and team communication and task management, there was recognition that it sometimes created burden for the patient and carers and that it was not always accurate in detecting health decline [53-55]. In line with recent reports, simplifying hardware and software use were deemed crucial for success [2].

The primary value proposition encompassed offering patients the option to select their care location and increasing access to rehabilitation without waiting in hospital for a bed. This was particularly pertinent for older individuals, considering the potential risks associated with hospital admissions [10], as well as for patients residing far from rehabilitation hospitals. Opinions on risk were varied; yet, overall, there was agreement that the VRW did not entail greater risks but rather presented different risks compared to inpatient rehabilitation. A notable concern was that patients reestablished contact with their community care teams, including GPs, a factor that could make cohesive care challenging. Additionally, fewer chances for in-person physical assessment, monitoring, and therapy raised concerns about clinicians possibly missing early signs of decline or patients receiving less intensive treatment compared to inpatient wards.

In terms of adopters, changing staff culture toward virtual care was recognized as an area for improvement. Many staff still preferred face-to-face visits due to familiarity or ease compared to dealing with technology. Uncertainty surrounding workflows and professional roles raised concerns that clinical staff were being asked to take on technology or administrative tasks. Furthermore, there were changes in staffing to align with the complexity of the patient cohort including the employment of a pharmacist and more experienced nurses. There was also a recognition of a shift in care responsibility to the patient and their family. Staff particularly acknowledged the crucial role that caregivers play in the patients’ journey during virtual care. Little is known about carers roles in virtual models [2], and our ongoing work is exploring the experiences of patients and their family members who were referred to the VRW.

Clear pathways for early recognition of patient deterioration and appropriate escalation procedures were in place to ensure patient safety (eg, patients having a single phone number to call and the availability of remote monitoring equipment). However, challenges arose from wider pressures on public Australian hospitals, including overcrowded emergency departments and insufficient ward beds, which hindered patient transfers. Patients were reluctant to return to the hospital for these reasons. This holds significance because prior research has indicated that a major concern among staff regarding virtual care is the ability to swiftly admit patients to the hospital if their condition deteriorates [56]. Interorganizational collaboration, particularly handovers with GPs, occurred infrequently, leading to difficulties in seamless patient care transitions.

To our knowledge, this study addresses a significant gap in understanding by delving into the perspectives of hospital staff regarding the facilitators and obstacles in implementing a VRW. A key strength of this research lies in its qualitative methodology, allowing for a deep exploration of the topic within its natural context and enabling a nuanced examination of the multifaceted social, cultural, and environmental factors at play. Moreover, by centering the voices and experiences of hospital staff, an often-marginalized group in research as evidenced by the paucity of published literature, we aim to provide a platform for their narratives to be heard and valued.

Virtual wards represent a relatively novel concept, and there exists some ambiguity regarding their terminology [2]. Subsequent studies might prioritize the global standardization of the model, recognizing the importance of precise terminology in ensuring the generalizability of insights within the literature. Despite these variations, our research findings retain applicability to services that use monitoring and virtual technologies for postdischarge patient care.

To summarize, most staff acknowledged the benefits of the VRW service but emphasized the need for sufficient resource allocation and additional time to plan and implement the service and address early hurdles. In terms of health care access and equity, it appears that in a rehabilitation setting with a primarily older cohort, technology complements physical consultations but does not replace them and this must be factored into service design and delivery. We underscore that new health care models cannot exist on the fringes of traditional frameworks, as they require their own well-defined structure and setup with clear workflow and professional responsibilities.

## Supplementary material

10.2196/54774 Multimedia Appendix 1 Interview questions.

10.2196/54774 Multimedia Appendix 2 Additional analysis information and researcher credentials.

10.2196/54774 Multimedia Appendix 3 Coding tree.

10.2196/54774 Multimedia Appendix 4 Additional quotes from hospital staff interviews.

## Abbreviations

COREQ: Consolidated Criteria for Reporting Qualitative Research
GP: general practitioner
NASSS: Nonadoption, Abandonment, Scale-up, Spread, and Sustainability
NHS: National Health Service
PRISM: Practical, Robust Implementation and Sustainability Model
RE-AIM: Reach, Effectiveness, Adoption, Implementation, and Maintenance
SALHN: Southern Adelaide Local Health Network
VRW: virtual rehabilitation ward

## References

[1] (2022). Tech-enabled virtual wards: relieving pressure on the NHS while caring for patients at home. NHS England.

[2] Norman G, Bennett P, Vardy ERLC (2023). Virtual wards: a rapid evidence synthesis and implications for the care of older people. Age Ageing.

[3] Best J (2022). The virtual wards aiming to ease hospital pressures. BMJ.

[4] Lewis G, Vaithianathan R, Wright L (2013). Integrating care for high-risk patients in England using the virtual ward model: lessons in the process of care integration from three case sites. Int J Integr Care.

[5] Toal D, Ryan A, Ryan K (2024). The response of hospital at home services during the COVID-19 pandemic: a scoping review. Home Health Care Management & Practice.

[6] (2022). Supporting information for ICS leads: enablers for success: virtual wards including hospital at home. NHS England.

[7] (2024). Virtual wards. NHS England.

[8] (2022). Bringing hospital care home: virtual wards and hospital at home for older people. British Geriatrics Society.

[9] Chua CMS, Ko SQ, Lai YF, Lim YW, Shorey S (2022). Perceptions of hospital-at-home among stakeholders: a meta-synthesis. J Gen Intern Med.

[10] Chen Y, Almirall-Sánchez A, Mockler D, Adrion E, Domínguez-Vivero C, Romero-Ortuño R (2022). Hospital-associated deconditioning: not only physical, but also cognitive. Int J Geriatr Psychiatry.

[11] Shepperd S, Butler C, Cradduck-Bamford A (2021). Is comprehensive geriatric assessment admission avoidance hospital at home an alternative to hospital admission for older persons?: a randomized trial. Ann Intern Med.

[12] (2021). Guidance note: frailty virtual ward (hospital at home for those living with frailty). NHS England.

[13] Hakim R (2023). Realising the potential of virtual wards. NHS Confederation.

[14] van Limburg M, van Gemert-Pijnen JEWC, Nijland N, Ossebaard HC, Hendrix RMG, Seydel ER (2011). Why business modeling is crucial in the development of eHealth technologies. J Med Internet Res.

[15] Greenhalgh T, Wherton J, Papoutsi C (2017). Beyond adoption: a new framework for theorizing and evaluating nonadoption, abandonment, and challenges to the scale-up, spread, and sustainability of health and care technologies. J Med Internet Res.

[16] Vindrola-Padros C, Singh KE, Sidhu MS (2021). Remote home monitoring (virtual wards) for confirmed or suspected COVID-19 patients: a rapid systematic review. EClinicalMedicine.

[17] James HM, Papoutsi C, Wherton J, Greenhalgh T, Shaw SE (2021). Spread, scale-up, and sustainability of video consulting in health care: systematic review and synthesis guided by the NASSS framework. J Med Internet Res.

[18] Sligo J, Gauld R, Roberts V, Villa L (2017). A literature review for large-scale health information system project planning, implementation and evaluation. Int J Med Inform.

[19] Greenhalgh T, Stramer K, Bratan T, Byrne E, Russell J, Potts HWW (2010). Adoption and non-adoption of a shared electronic summary record in England: a mixed-method case study. BMJ.

[20] Greenhalgh T, Robert G, Macfarlane F, Bate P, Kyriakidou O (2004). Diffusion of innovations in service organizations: systematic review and recommendations. Milbank Q.

[21] Hunter W (2022). Virtual wards: a bridge between hospitals and the community?. Nursing in Practice.

[22] Sheasby L Virtual ward challenges and how to overcome them. The Access Group.

[23] Hutchings R, Edwards E (2023). Virtual wards: the lessons so far and future priorities. Nuffield Trust.

[24] Guba EG, Lincoln YS (1989). Fourth Generation Evaluation.

[25] Dyb K, Berntsen GR, Kvam L (2021). Adopt, adapt, or abandon technology-supported person-centred care initiatives: healthcare providers' beliefs matter. BMC Health Serv Res.

[26] Thomas EE, Taylor ML, Ward EC (2024). Beyond forced telehealth adoption: a framework to sustain telehealth among allied health services. J Telemed Telecare.

[27] Abimbola S, Patel B, Peiris D (2019). The NASSS framework for ex post theorisation of technology-supported change in healthcare: worked example of the TORPEDO programme. BMC Med.

[28] Glasgow RE, Vogt TM, Boles SM (1999). Evaluating the public health impact of health promotion interventions: the RE-AIM framework. Am J Public Health.

[29] Feldstein AC, Glasgow RE (2008). A practical, robust implementation and sustainability model (PRISM) for integrating research findings into practice. Jt Comm J Qual Patient Saf.

[30] Tong A, Sainsbury P, Craig J (2007). Consolidated criteria for reporting qualitative research (COREQ): a 32-item checklist for interviews and focus groups. Int J Qual Health Care.

[31] CC BY 4.0 deed. Creative Commons.

[32] (2022). Snapshot of South Australia - high level summary data for South Australia in 2021. Australian Bureau of Statistics.

[33] Flavel J, Baum F, Musolino C, Freeman T, van Eyk H, Southgate Institute for Health, Society and Equity (2020). Healthy south: population health and social determinants in Southern Adelaide. Flinders University.

[34] Luciani M, Campbell K, Tschirhart H, Ausili D, Jack SM (2019). How to design a qualitative health research study. part 1: design and purposeful sampling considerations. Prof Inferm.

[35] Creswell JW, Poth CN (2016). Qualitative Inquiry and Research Design: Choosing Among Five Approaches.

[36] Patton MQ (2014). Qualitative Research & Evaluation Methods: Integrating Theory and Practice.

[37] Starks H, Trinidad SB (2007). Choose your method: a comparison of phenomenology, discourse analysis, and grounded theory. Qual Health Res.

[38] Hamm LM, Boluk KA, Black JM, Dai S, Thompson B (2019). Phenomenological approach to childhood cataract treatment in New Zealand using semi-structured interviews: how might we improve provision of care. BMJ Open.

[39] Buys T, Casteleijn D, Heyns T, Untiedt H (2022). A reflexive lens on preparing and conducting semi-structured interviews with academic colleagues. Qual Health Res.

[40] Ritchie J, Spencer L, Bryman A, Burgess B (1994). Analyzing Qualitative Data.

[41] Gale NK, Heath G, Cameron E, Rashid S, Redwood S (2013). Using the framework method for the analysis of qualitative data in multi-disciplinary health research. BMC Med Res Methodol.

[42] Iliffe S, Wilcock J, Drennan V (2015). Changing practice in dementia care in the community: developing and testing evidence-based interventions, from timely diagnosis to end of life (EVIDEM). Programme Grants Appl Res.

[43] Olmos-Vega FM, Stalmeijer RE, Varpio L, Kahlke R (2022). A practical guide to Reflexivity in qualitative research: AMEE guide no. 149. Med Teach.

[44] Walsh R (2003). The methods of reflexivity. The Humanistic Psychologist.

[45] Parkinson S, Eatough V, Holmes J, Stapley E, Midgley N (2016). Framework analysis: a worked example of a study exploring young people’s experiences of depression. Qualitative Research in Psychology.

[46] Pope C, Ziebland S, Mays N (2000). Qualitative research in health care. analysing qualitative data. BMJ.

[47] Collins B (2015). Staff engagement: six building blocks for harnessing the creativity and enthusiasm of NHS staff. The King’s Fund.

[48] Sharma U, Clarke M (2014). Nurses' and community support workers' experience of telehealth: a longitudinal case study. BMC Health Serv Res.

[49] Wade VA, Eliott JA, Hiller JE (2014). Clinician acceptance is the key factor for sustainable telehealth services. Qual Health Res.

[50] Mohammed HT, Hyseni L, Bui V (2021). Exploring the use and challenges of implementing virtual visits during COVID-19 in primary care and lessons for sustained use. PLoS One.

[51] Zhang X, Saltman R (2022). Impact of electronic health record interoperability on telehealth service outcomes. JMIR Med Inform.

[52] Callisaya ML, Lee AHC, Khushu A (2021). Rapid implementation of telehealth in geriatric outpatient clinics due to COVID-19. Intern Med J.

[53] Talbot B, Farnbach S, Tong A (2022). Patient and clinician perspectives on the use of remote patient monitoring in peritoneal dialysis. Can J Kidney Health Dis.

[54] Thomas EE, Taylor ML, Banbury A (2021). Factors influencing the effectiveness of remote patient monitoring interventions: a realist review. BMJ Open.

[55] Primholdt Christensen N, Skou KE, Boe Danbjørg D (2021). Health care professionals' experiences with the use of video consultation: qualitative study. JMIR Form Res.

[56] Thornton N, Horton T, Hardie T (2023). How do the public and NHS staff feel about virtual wards?. The Health Foundation.

