# Supplementary material for: Hospital Staff Perspectives on the Drivers and Challenges in Implementing a Virtual Rehabilitation Ward: Qualitative Study
Source: JMIR Aging. 2024 Jun 27;7:e54774. doi: 10.2196/54774 (PMC11220728; doi:10.2196/54774)
Supplement: Multimedia Appendix 1 [file aging-v7-e54774-s001.docx]

| **NASSS-CAT Domain** | **Domain Description** |
| --- | --- |
| Nonadoption: | This domain refers to the factors that hinder the initial adoption of a technology in a healthcare setting. These factors could include financial constraints, lack of resources, or resistance from stakeholders. |
| Abandonment: | This domain refers to the factors that lead to the discontinuation of a technology after it has been implemented. These factors could include technical difficulties, poor usability, or lack of user engagement. |
| Scale-up: | This domain refers to the factors that facilitate the expansion of a technology from a small pilot program to a larger, more widespread implementation. These factors could include favourable regulatory policies, strong leadership, or effective dissemination strategies. |
| Spread: | This domain refers to the factors that enable the successful diffusion of a technology across different healthcare settings, populations, and contexts. These factors could include adaptability, interoperability, or cultural sensitivity. |
| Sustainability: | This domain refers to the factors that ensure the long-term viability and impact of a technology in a healthcare setting. These factors could include organizational support, ongoing training, or continuous improvement. |

**Multimedia Appendix 1. Interview questions.**

| **NASSS-CAT Domain** | **Interview Question** | **Prompt** |
| --- | --- | --- |
| Introduction | - What is your role and how long have you worked for the Virtual Rehab team? |  |
| **Condition**   - Nature of condition or illness - Comorbidities - Sociocultural factors | - Are there any populations where you think virtual rehabilitation does not work? - Do you think virtual rehabilitation is servicing patients from diverse cultural backgrounds? - What has been your experience working with these populations in this service? - Are there other groups who might not be accessing the service and if so, why? - Was it possible to provide evidence-based guidelines care (e.g. stroke, hip fracture) to each patient? |  |
| **Technology**   - Material properties - Knowledge to use it and acceptability - Knowledge generated by it - Supply model - Performance and dependability - Technical interdependencies | - What technologies are used? How would you rate the technology’s performance and dependability? - Anything that could be improved? - How was the information gained from monitoring used? - What is the main troubleshooting that takes place? |  |
| **Value proposition**   - Supply-side value (to developer) - The value to the healthcare system (e.g. efficacy) is uncertain - Demand-side value (to consumer) - The value to the intended users (e.g. patients, clinicians) is uncertain | - Can you tell me how you understand risk? - What risks do you think there are in remote consulting? - What risks do you think there are in remote monitoring? - What experiences have you had where remote consulting felt less safe than in ED? - Do you have views on what might reduce the risk in remote consulting? - Do you have any examples of where remote care resulted in overtreatment or undertreatment? - Did you always have access to patient records? - Do you think the patients were always aware of your name and role? Or did you feel they were confused? - What about prescribing – were you confident you had a safe system in place to prescribe and dispense medications? Are there any examples of problems? - Do you have access to the resources and equipment you need to treat patients effectively and safely? - Did any of your patients have to be admitted to hospital or transferred to the FMC ED? If so, tell me about this. - Was making the decision to transfer difficult? What are the key factors you think about when transferring? - Was the transfer process difficult? Why? |  |
| **Adopters**   - Staff (role, identity) - Patient (passive vs. acute input) - Carers (available, type of input) | - Would you prefer to see patients face to face? - Did you feel able to build rapport/trust with patients and families when providing care remotely? - Tell me about how your current role differs from previous roles? - Thinking about last week, did you have to do tasks for your patients that you would not do when working on the wards? What tasks? Prompts – e.g., organise medical appointments/imaging, take them to medical appointments, liaise with health care workers etc. - Do you think this is part of your job or could someone else have done this? If so, whom? |  |
| **Organisations**   - Capacity to innovate in general - Readiness for this technology - Nature of adoption and/or funding decision - Extent of change needed to organisational routines - Work needed to plan, implement, and monitor change | - Have the technology’s benefits to the clinician been shown to outweigh the hassle of using it? - Are there areas where you should have received more training when commencing this rotation? - Are there areas where you feel you still need more training? | - To what extent would implementing the technology require staff to do their jobs in a different way and/or interact with different people or teams? - To what extent would implementing the technology require new or different steps in the care pathway (e.g. new administrative processes)? |
| **Wider system**   - Political/policy context - Regulatory/legal issues - Professional bodies - Sociocultural context - Interorganisational networking | - Did you participate in any telehealth case conferences where the GP was included in the meeting? - [If did participate in case conferences] Tell me about these? - Does this happen routinely? - To what extent do you think this technology implies major changes to the way healthcare is delivered? | - How well do you think the case conferences went? - How useful do you think the case conferences were? - Were there any problems with the case conferences? (eg GP not available, actions not followed through, tech problems) |
| **Embedding and adaptation over time**   - Scope for adaption over time - Organisational resilience | - What are three key skills someone working in the virtual rehab team needs? - In terms of delivering virtual rehabilitation to patients, can you suggest any improvements that could be made to the care process? | - With the benefit of hindsight, if you were designing the virtual rehabilitation service, are there things you would do differently than now? |

**Note:** This was a small-scale and exploratory study with a finite number of participants, so we decided to proceed directly to the main data collection phase and not to conduct a pilot study. No repeat interviews were carried out.
