# Supplementary material for: Hospital Staff Perspectives on the Drivers and Challenges in Implementing a Virtual Rehabilitation Ward: Qualitative Study
Source: JMIR Aging. 2024 Jun 27;7:e54774. doi: 10.2196/54774 (PMC11220728; doi:10.2196/54774)
Supplement: Multimedia Appendix 2 [file aging-v7-e54774-s002.docx]

**Multimedia Appendix 2. Additional analysis information and researcher credentials.**

| **Stage** | **Task** | **Process** |
| --- | --- | --- |
| 1 | Familiarization | Anonymized transcripts were uploaded to NVivo (QSR International) for the familiarization process. This stage allowed MR to become familiarized with the data and LG, who conducted the interviews, to gain a deeper familiarization of the written data. This stage also sensitized the researchers to early themes that were present in the data. We made a conscious effort to be mindful of individual differences in the data as these often dissolve when coding commences [42]. This focus was important because we wanted to identify within- and between-participant differences to determine if, for example, differences in discipline or job level (i.e. junior vs. senior) had an impact on experiences or views. Researchers could refer to the original audio recordings if needed, to gain a deeper understanding of the conversation. The researchers utilized a practical guide to reflexivity in qualitative research [43] which recommended Walsh’s methods [44]. Walsh’s model has four overlapping and interacting elements: personal, interpersonal, methodological, and contextual. Researchers also kept memos to document initial impressions of the raw data, for example, where staff opinions significantly differed on a topic. |
| 2 | Coding | Three random transcripts were chosen for initial coding. The transcripts were printed with large margins for note taking. Working separately, LG and MR identified key themes, issues, or discussion items and assigned a code that captured the essence of the point. Codes were both deductive, using the Nonadoption, Abandonment, Scale-up, Spread, and Sustainability (NASSS) framework and inductive. Researchers used written notes to record ideas relevant to the future analysis. |
| 3 | Identifying an analytical framework | An analytical framework can be defined as a set of codes that have been arranged into categories that will be used to manage and organize data [41]. Categories refer to codes that have been grouped together based on similarities. This stage is based around ‘sifting and sorting’ rather than making sense or understanding the whole data set which occurs during stage five [45]. Although categories are closely linked with the raw data, the development of categories is a step towards the abstraction of data, that is, the process of explaining the data as a whole rather than looking at it from individual anecdotes [41]. After we had coded the original three transcripts, we met to discuss the codes, keeping in mind how it informed our research question. A third reviewer was available if there were disagreements, but this was not needed. As we utilized the NASSS framework [15] to design our interview schedule, a-priori categories were already established (deductive code identification), and these formed the basis for creating our original overarching categories and emergent codes. However, there was also scope to inductively establish codes during this stage, although, for this study, no inductive codes were created as the data collected aligned with seven NASSS domains. Once we had agreed on codes and a brief definition of what each code represented, this formed the basis of our analytical framework. We then independently coded three further transcripts in NVivo using the initial framework and noted any new codes which did not fit the existing set. We met again to discuss and refine codes. Codes that were conceptually related were reduced, summarized, and grouped around related categories. This iterative process of applying and refining the analytical framework was repeated until no new codes or categories were produced. |
| 3 | Indexing into categories | During the third stage, we systematically applied the codes from our analytical framework to all our data sets in NVivo. This is also where we started data abstraction by assigning higher-level categories which guided us during stage four. We aimed to construct a detailed index of the data, providing us with ‘manageable chunks for subsequent retrieval and exploration’ [46]. |
| 4 | Charting and summarizing | This stage required us to order and abstract the indexed study data so it could be examined systematically as a whole. In NVivo, we grouped codes and corresponding higher-level categories within a matrix. The matrix was set out so that each row represented a participant, and each column represented a code. We then used the autocode function in NVivo which abstracted individual participant data from each transcript for each code, summarizing the point using verbatim words. Quotes that were particularly pertinent were highlighted in colour within the matrix. |
| 5 | Mapping and interpretation | LG and MR reviewed the matrices, looking for connections between and within participants and categories to generate overall themes from the data. This stage was influenced by our a-priori research question. We attempted to explain overall themes within the data as a whole, rather than focusing on individual cases. This was achieved through our memos and discussions. |

| **Researcher** | **Researcher Credentials** | **Researchers Occupation** | **Sex** | **Training** | **Researcher Characteristics** |
| --- | --- | --- | --- | --- | --- |
| LG | PhD | Researcher | Female | - BSc Psychology - MSc Applied Cognitive Neuroscience - PhD Neuropsychology - Numerous post-doc roles on qualitative aged care projects | - Experience caring for older family members with dementia within the UK healthcare system - Previously worked as an Assistant Psychologist in National Health Service (NHS). - Employed in the same department that received the funding to establish and evaluate the new rapid assessment frailty service for older adults. |
| MR | PhD | Researcher | Female | - BSc Occupational Therapy - PhD aged care - Extensive experience as a post-doc researcher on aged care projects | - Previously worked in Australian health service as an Occupational Therapist. - Employed in the same department that received the funding to establish and evaluate the new rapid assessment frailty service for older adults. |

**Note:** Neither of the researchers have worked for or experienced care from a virtual ward and, therefore, may have different perspectives from the healthcare staff implementing the service. However, researchers shared characteristics with staff recruited to the study such working within public healthcare systems and the complexity around this. Neither of the researchers were frequent users of rehabilitation services but all had prior personal or family experience with such services. Both researchers have an interest in aged healthcare research, and all were aware of the strains on the national and international healthcare systems.

The participants in the study knew that LG was an academic researcher from the UK who was working on the evaluation project for the Virtual Rehabilitation Ward as part of the University of Flinders Rehabilitation, Aged and Palliative Care team.
