# Supplementary material for: Hospital Staff Perspectives on the Drivers and Challenges in Implementing a Virtual Rehabilitation Ward: Qualitative Study
Source: JMIR Aging. 2024 Jun 27;7:e54774. doi: 10.2196/54774 (PMC11220728; doi:10.2196/54774)
Supplement: Multimedia Appendix 3 [file aging-v7-e54774-s003.docx]

| Agreed themes for analytical framework |
| --- |
| Theme 1: Condition   - 1. Nature   2. Comorbidities   3. Sociocultural factors |
| Theme 2: Technology  2.1 Integrated care systems and service providers  2.2 Education and knowledge  2.3 Usability and dependability  2.4 Benefits and barriers |
| Theme 3: Value proposition  3.1 Choice in care location  3.2 Treatment at home  3.3 Empowering patients  3.4 Risk |
| Theme 4: Adopters  4.1 Staff culture change  4.2 Workflows and staff skill set  4.3 Burden on patient and carer |
| Theme 5: Organizations  5.1 Identity of the service  5.2 Explaining the Virtual Rehabilitation Ward  5.3 Resource allocation |
| Theme 6: Wider system  6.1 Fill gaps with hospital pressures  6.2 Patients want to be at home  6.3 Continuity of care  6.4 Interorganizational working  6.5 Transferring back to hospital |
| Theme 7: Embedding and adaptation over time  7.1 Advantages  7.2 Planning  7.3 Refinement  7.4 Sense-making  7.5 Reflection  7.6 Adaption |

**Multimedia Appendix 3. Coding tree.**
