# Supplementary material for: Hospital Staff Perspectives on the Drivers and Challenges in Implementing a Virtual Rehabilitation Ward: Qualitative Study
Source: JMIR Aging. 2024 Jun 27;7:e54774. doi: 10.2196/54774 (PMC11220728; doi:10.2196/54774)
Supplement: Multimedia Appendix 4 [file aging-v7-e54774-s004.docx]

**Multimedia Appendix 4. Additional quotes from hospital staff interviews.**

| **NASSS Domain** | **Key message** | **Additional Quotes** |
| --- | --- | --- |
| **1. The Condition** | - High level care and some conditions more challenging to manage - Requires consideration of comorbidities - Sociodemographics and inability to use technology is a barrier | *we are taking patients that otherwise would be inpatients in hospital and therefore they've got multiple medical problems so they can get unwell and then you are always just thinking where are they best cared for?* (Doctor)  *And some of them are blind, so they can't see, so that makes it hard to navigate the iPad, and some of them have sensation changes in their fingertips, they can't actually feel how heavy they're pressing.* (Allied Health)  *I think that the age and demographics that we work with isn't very conducive to the virtual first approach at times. I think lots of our patients are older and they really struggle with technology. They get really overwhelmed quite easily. And they're already in an overwhelmed state from a medical point of view, from being in hospital and just going home, and then to add that on top of it, it can be really hard.* (Allied Health) |
| **2. The Technology** | - Poor interoperability and delays in pinpointing problems impedes adoption - Technology is useful for escalation of care, communication, and task management - Barriers with dependability and usability of technology - Education around use of technology is important | *we've had to update the various iPads and the computers and things like that to try and fix these bugs and problems...but all the iPads are scattered at all these different people's houses...So it's a little bit clunky updating them.* (Doctor)  *So, with all those cogs there, I'd have to say that there's a lot of finger pointing as to who is at fault for something not working. And ultimately, who suffers is that the patient doesn't get the best service, and our clinicians get frustrated by the system.* (Leadership/Admin Team)  "This is ridiculous." It's frustrating for everyone. I might just do home visits instead*.* (*Allied Health)*  *Medtasker is a way that we can communicate to the doctors, and the wider team, similar to what you'd use the CAT board on the wards…it's actually good. Yeah. I was quite excited for that because I was used to having something similar on the wards.* (Allied Health)  *I think if you are comparing it to sending people home and doing phone reviews of patients without the monitoring or without the video calls, I think it's definitely worth it. I think being able to see a patient and see if they look well or unwell and what they're actively able to do as well as being able to see trends in their observations and blood pressure and all of those kind of things to help guide your medical management. (*Doctor)  *It just, hasn't been as good as what we had expected as far as patient usability is concerned, how fresh it looks, and how attractive it looks for the patient, and for the clinician, and the fact that it's very slow.* (Leadership/Admin Team)  *So instead of naming the app that sometimes sound a bit complex, I'll say, "There's the purple app that we use when we are going to connect a call and the green app is what we use when we monitor you."* (Nurse)  *And it's hard to build a rapport with someone when you're making them do something they already don't want to do, like use an iPad. And then when it doesn't work, obviously their tolerance is quite low. I think it really impacts on the clinician, the relationship and rapport that you build with them.* (Allied Health)  *Some [staff] think it's a complete waste of time. And I was thinking that, but some of it can be put back on us as well, not using the technology how it was supposed to be used. Something that can go back to training, and also how we deliver our training to the patients...So I think we've got to take some ownership in some of the failures as well. We can't just point the finger at [telecommunications company].* (Leadership/Admin Team) |
| **3. Value Proposition** | - Promotes choice and self-management for patients - Enables access for those in remote communities - Different risks to inpatient wards | *Especially those with cognitive issues, a change of environments, they're too noisy, they're too loud, they're too bright, too clinical. It's just too upsetting.* (Leadership/Admin Team)  *I think in the patient's best interest to have the option. Lots of people don't want to actually drive into [hospital], find a park, pay their parking fee...It's all confusing for them...But there might be patients that are just like, "I hate technology, I've always hated technology, I'm not going to touch it. I'd prefer to come in person." It's a patient preference...we can genuinely offer what's the best for them.* (Doctor)  *It really promotes self-management and equipping them with the skills to know what to do and how to position themselves and what to consider and how to keep each other safe. I think that would carry on after our services done. They've been trained, they've been upskilled, they're involved in the process, so they'll probably take those skills forward into the future.* (Allied Health)  it's good to be able to see people that live further away, or out of catchment, which we wouldn't be able to see if we didn't have telehealth.  *Or, you simply don't challenge them quite as much...subtherapeutic is probably, is more likely, and there are times when that happens.* (Allied Health) |
| **4. The Adopters** | - Staff culture change required regarding virtual care - Importance of workflows and staff skill set - Shift of burden from staff to patient and family | *I think it's a mindset issue. It's just, 'If I can see them in person, I will.' That's what they've learned to do.* (Leadership/Admin Team)  *I think it should have been part of the tele-rehab role or admin. You know what I mean? I think my nurses should just take the kit out if needed, and educate, and basically be nurses.* (Leadership/Admin Team)  *when we first recruited to virtual rehab, we had a cohort of assistants in nursing, and clinical nurses. We soon realized that assistants in nursing didn't work for us, because of the scope of practice.* (Leadership/Admin Team)  *Given that it's community service and given that our patients are very medically unwell, juniors need a lot of support and supervision…a lot of safety netting because a lot of things can go wrong.* (Allied Health)  *I think some of the logistics take a lot of time, and things like transport, and... Just to appointments, or to scans, or different ways of trying to keep people at home. It's just a lot of discussion, and a lot of phone calls and things at times. So it can take a while.* (Doctor)  *I think some patients weren't appropriate for iPad reviews. And then you'd talk to the family a bit more.* (Allied Health) |
| **5. The Organisation** | - Clarity about the identity of the service and stricter triage - Explaining the virtual rehabilitation ward to patients - Resource allocation | *when we came into this ward, we thought virtual rehab, and I was actually even telling staff, that we would be doing a lot more things virtually. We wouldn't be going and having as much face-to-face contact. The thing that's actually turned out, is nurses are having mostly face-to-face contact. And that's because of the cohort of patients that we are taking.* (Leadership/Admin Team)  *And I think a lot of the patients... Not get confused. I think there's a bit of a grey area between, because we're technically a ward, they get off a lot more lightly than if they were in a ward, but it's often a lot more than they expect. I don't think they fully understand. They think it's just going to be a couple visits a week. They don't understand that, yes, you're going home but it's still very intense. So I think that gets lost in translation as well.* (Leadership/Admin Team)  *Yeah, sometimes there is a lack of computers, especially since we've gotten a bit more staff as well. Always fighting for a computer, so that's been an issue lately as well.* (Allied Health)  *…when we do video calls or huddles in those little offices, other people are making phone calls as well. I mean, we do have noise cancelling headphones, but they're not really noise cancelling.* (Allied Health)  *we've got five cars and three of them are allocated to nursing. So that leaves two cars for Allied Health and at any one day there would be at least probably 10 Allied Health clinicians on.* (Allied Health) |
| **6. The Wider Context** | - Trying to fill gaps and hospital pressures - Patients want to be at home - Continuity of care and interorganisational working - Transferring back to hospital | *I think it means that we can step people down into their own homes a little bit more. And if we had a good streamlined and robust way of doing it, I think that it would free up more beds in that subacute space in the hospital. (*Doctor)  *On the whole, I think patients really enjoy it because they're in home and we are coming to them or virtually. It's a positive thing.* (Nurse)  *…because they're in the community, it's not easy to just get them back in through our radiology. (Doctor)*  *Although they're technically an impatient, we do try to get them set up with a GP appointment, we just want them to start, to bring the GP into the picture early so that their GP knows what's happening…Then we try with the nursing and medical monitoring to really catch things early so that we can try and avoid these up transfers or representation to ED.* (Doctor) |
| **7. Embedding and Adaptation Over Time** | - Advantages for patients, clinicians and healthcare system - Need for planning a virtual service - Refinement - Sense making, collective reflection, and adaptive action | *So, I find that the ability to connect with patients via telehealth is so useful, and I think there's a lot of benefits to it. I mean, I think the patients appreciate being able to be in touch. (*Doctor)  *I think they are often much more relaxed in their own environment, and I found that most of the patients are appreciative of the phone call and the ability to be able to connect to doctors and staff so easily.* (Doctor)  *I think it's a great idea. I think it's a little bit ahead of its time and the tech's not there and the patients are not quite the right demographic for it. But I think it's a really good service. I think it's beneficial. I think it's important.* (Allied Health)  *physically I think the space has been an issue, so the registrars and the RMOs on two different levels and we are somewhere else, so it's a lot of liaising and communicating, and those incidental conversations and planning may not necessarily happen, be happening.* (Doctor)  A*nd we're an evolving service too. Our experience is evolving.* (Nurse)  *But I think the concept is necessary and it's time for someone to start figuring out how it all works and you don't know how it's going to work until someone does it. I think it has to be like a guinea pig service to figure it out, and yeah, I think it's good.* (Leadership/Admin Team)  *But we, yeah, we've kind of changed our models and our streams as we've gone along. So I guess, it's not clear what our identity is, and how to best send that message to any stakeholders.* (Leadership/Admin Team) |
